# Effectiveness of Peer Support Programs for Severe Mental Illness: A Systematic Review and Meta-Analysis

**DOI:** 10.3390/healthcare12121179

**Published:** 2024-06-11

**Authors:** Sung-Nam Lee, Hea-Jin Yu

**Affiliations:** College of Nursing, Sahmyook University, Seoul 01795, Republic of Korea

**Keywords:** severe mental illness, peer support, meta-analysis, supervision

## Abstract

(1) Background: While medication and various forms of psychotherapy are common treatments for severe mental illness, peer support programs have also proven to be effective in managing mental disorders. These programs, which involve individuals with similar experiences in navigating mental health challenges, aim to improve coping skills and foster supportive community networks. However, despite the prevalent mention of peer support programs, especially those with supervision, there has been no systematic review or meta-analysis of peer support supervision. This study aimed to systematically review and meta-analyze the forms and effectiveness of peer support programs for individuals with severe mental illnesses. (2) Methods: A literature search focusing on randomized controlled trials (RCTs) published between February 2003 and January 2024 was conducted. (3) Results: Sixteen RCTs meeting the inclusion criteria and involving a total of 4008 participants were reviewed. These studies utilized various peer support program strategies, with eight studies included in the qualitative analysis. The combined effect sizes for depressive symptoms (d = 0.12; 95% CI, −0.14, 0.37; *p* = 0.37), empowerment (d = 1.17; 95% CI, −0.81, 3.15; *p* = 0.25), quality of life (d = 0.70; 95% CI, −0.12, 1.52; *p* = 0.09), psychiatric symptoms (d = −0.05; 95% CI, −0.20, 0.10; *p* = 0.54), and self-efficacy (d = 0.20; 95% CI, 0.05, 0.36; *p* = 0.01) were assessed. (4) Conclusions: Our analysis emphasizes the need for further studies on peer support programs for individuals with severe mental illness, particularly those focused on self-efficacy outcomes across diverse geographic locations involving more countries and with larger scales to bolster the strength of the evidence.

## 1. Introduction

Severe mental illness (SMI) is defined as a subset of illnesses that produce psychotic symptoms, including schizophrenia spectrum disorders, depression, and bipolar disorder [1]. Globally, approximately 20 million people (0.3%) have schizophrenia, 46 million (0.6%) have bipolar disorder, and 264 million (3.4%) have major depression [2]. SMI is debilitating; patients face difficulties with their daily living activities and struggle with social and functional impairments [1]. More seriously, individuals with SMI exhibit a marked 10–20 year diminished average life expectancy compared with their healthy counterparts. Furthermore, their premature mortality rate is three times greater than that of the general population, even within age-matched peer groups [3,4].

The majority of individuals with SMI continue to utilize mental health services for an average of 13 years and experience multiple acute psychiatric admissions [5]. Those with SMI often exhibit resistance to conventional treatments due to difficulties in managing daily activities [4], leading to various functional disabilities, particularly in the social and occupational domains [6,7]. To address these challenges, modern mental health systems have transitioned from a healthcare model to a recovery-oriented one [8].

Owing to disparities in perspectives between clinicians and consumers/clients regarding mental illness, recovery can be broadly categorized into personal and clinical recovery [9]. The former is framed in terms of subjective definitions (e.g., journey, process, and non-linearity), whereas the latter is defined in terms of clinical–functional aspects (e.g., function, ability, and symptom outcomes). Personal recovery is seen as complementary to clinical recovery, emphasizing processes rather than outcomes [8,10,11]. Five key concepts elucidate personal recovery: connectivity (establishing positive relationships), hope (maintaining optimism about the future), identity (fostering positive self-esteem), meaning (finding purpose in life), and empowerment (exercising self-regulation and control) [12]. In essence, recovery is an intensely personal and individualized journey that transforms a person’s attitudes, values, emotions, goals, skills, and/or roles to live a fulfilling, optimistic, and contributory life despite the constraints imposed by illness [8].

With increasing advocacy for personal recovery, peer-delivered self-management interventions have garnered growing enthusiasm as a complementary treatment to traditional care [13]. Peer support entails psychosocial rehabilitation wherein individuals with mental health issues share their recovery experiences with others facing similar challenges, thus engaging in a reciprocal process of giving and receiving help based on mutual respect and shared responsibility [11,14]. Peer support can be administered in various settings, either as an independent service or as an integral component of professional care [15].

Previous research has demonstrated the positive impacts of peer support services on mental symptoms, social functioning, and life satisfaction among individuals with SMI [11,16,17]. Alongside improvements in functioning, recovery, social networks, and employment opportunities for peer support workers [18,19], the results of other studies have shown enhancements in employee attitudes toward service users, the adoption of recovery-oriented approaches, reduced hospitalization rates, and various recovery outcomes [17,19,20,21]. However, some prior RCTs comparing peer support services with conventional care have reported either no difference in the effectiveness of the two groups [22] or that peer support services are less effective [23,24]. In a meta-analysis of previous RCTs, Lloyd-Evans et al. [25] found minimal overall positive effects on clinical recovery but observed some evidence of symptom relief contributing to individual recovery. Results from a recent systematic review and meta-analysis by White et al. suggest potential positive impacts on psychosocial outcomes but indicate unlikely improvements in clinical outcomes [26]. Lyons et al. proposed that their interventions could lead to slight enhancements in overall recovery; however, their findings suggest they may not specifically address improvements in individual hope or empowerment, and they do not seem to alleviate clinical symptoms [27].

Lloyd-Evans’ study [25] faced a high risk of bias, exacerbated by variability in participant characteristics and program content, making it challenging to pinpoint factors that may have influenced the results. White [26] investigated one-on-one peer support interventions for adults using mental health services and included several pilot studies with a small number of participants, whereas Lyons [27] focused solely on group peer support interventions. Such findings highlight the heterogeneity in program characteristics and methodological flaws within the current evidence base, raising questions about the effectiveness of peer support services [26,27,28].

Currently, systematic reviews of peer support services are underway for each type, with the integration of peer support service types reduced from six to four types [29]. However, due to the limited disclosure of peer support service contents or types in the literature and the dearth of similar RCT studies, conducting a meta-analysis of identical services is challenging. Despite the prevalent mention of peer support supervision in methodology sections, there has been no systematic review or meta-analysis focusing specifically on peer support supervision. Peer support, as a model, promotes diversity and individual strengths while emphasizing mutual respect, shared responsibility, and consent to aid. Peer supervision is crucial for operationalizing these principles effectively [11].

Supervision has been recognized as vital for the successful integration of peer specialists into community mental health settings [30,31,32]. Thus, this study aims to establish a foundation for effective peer support service implementation through a systematic review and meta-analysis of the relationship between peer support service outcomes and the supervision peer providers receive. We also examine how peer-assisted supervision in both one-on-one and group contexts contributes to intervention heterogeneity.

The aim of this systematic review and meta-analysis was to evaluate randomized controlled trials (RCTs) assessing the effectiveness of peer support programs for individuals with SMI. The specific aims were as follows: (1) identify the types of peer support programs utilized in outpatient or inpatient settings for individuals with SMI; (2) determine the primary outcomes of these peer support programs for individuals with SMI.

## 2. Materials and Methods

### 2.1. Eligibility Criteria

Eligible participants were adults aged 18 and above diagnosed with SMI. Confirmation of mental health conditions among participants was based on meeting specified criteria. Studies were included if peer support was intentionally provided by a peer worker for adults using mental health services. Studies published within the last 20 years (from February 2003 to January 2024) were considered.

Exclusions comprised studies where peer support provided by a peer worker was not the primary intervention method and those where mental health was not the primary focus of the intervention. The other exclusion criteria were studies not in English; studies involving participants solely with personality disorders, organic neuropathology, or disorders related to alcohol or substance abuse; studies involving only inpatients; and unpublished papers or dissertations.

### 2.2. Study Design

The research methodology employed in this study is a systematic review and meta-analysis focused on RCTs and published trials while excluding other study types.

### 2.3. PICO (Population, Intervention, Comparison, Outcome)

#### 2.3.1. Population

Adults aged 18 and above diagnosed with SMI.

#### 2.3.2. Intervention

The included studies consisted of RCTs exploring various interventions, including peer-delivered self-help, peer-run services, peer partnerships, and the employment of individuals in recovery as peers.

#### 2.3.3. Comparator

Studies with a comparator included usual care or waitlist control groups.

#### 2.3.4. Outcomes

Included studies reported outcomes related to personal recovery such as hope, identity, personal confidence, self-efficacy, quality of life, relationships, empowerment, and working alliance. Clinical recovery outcomes were also considered, encompassing studies reporting clinical outcomes (e.g., any measure of psychiatric symptoms), including clinical recovery rates.

### 2.4. Search Strategy

#### 2.4.1. Search Methods

The literature review process adhered to the guidelines outlined by the Cochrane Collaboration for systematic reviews of interventions [33] and followed the Preferred Reporting Items for Systematic Reviews and Meta-Analysis (PRISMA) statement [34]. The review protocol was officially registered in INPLASY (International Platform of Registered Systematic Review and Meta-analysis Protocols) with registration ID INPLASY202430127. A comprehensive search was conducted on PubMed, Embase, Cumulative Index to Nursing and Allied Health Literature (CINAHL), and the Cochrane Library. Additionally, hand-searching was performed using the Google Scholar electronic database.

Searches encompassed the past 20 years, from February 2003 to January 2024, with no alterations to search terms or strategy (Supplementary Material Table S1). The literature search terms were based on those used in MEDLINE and tailored to each database’s characteristics. The search terms were combined using Boolean operators (OR and AND). MESH (medical subject heading) terms were initially identified, with participant (P) terms consisting of “mental disorder”, “psychiatric disorder”, “mentally ill persons”, “mentally disabled”, and “severe mental illness” (I) combined with terms like “peer counselor”, “peer provider”, “peer educator”, “peer specialist”, “consumer advocator”, “peer support programs”, “psychosocial interventions”, “peer-delivered intervention”, “mutual support”, “self-help groups”, and “organization AND administration”. To clarify, we utilized the terms “organization” and “administration” to denote “supervision”, which are MESH terms corresponding to supervision.

#### 2.4.2. Study Selection and Data Extraction

All retrieved papers were imported into an Endnote library, and duplicates were manually removed using software. Two reviewers (SNL and HJY) independently evaluated titles and abstracts based on the predefined eligibility criteria. Discrepancies during title and abstract reviews were resolved through discussions between the two reviewers. Studies not meeting inclusion criteria were excluded at this stage.

#### 2.4.3. Assessment of Risk of Bias

Risk of bias assessment in the selected literature was independently conducted by two review authors (SNL and HJY) using the Cochrane Group’s Randomized Controlled Trials Assessment Tool, Cochrane Risk of Bias (ROB), version 5.4.1. The researchers deliberated on any disagreements in assessment outcomes until a consensus was reached. Bias risk for each domain was categorized as high (indicating significant undermining of result confidence), low (suggesting minimal impact on results), or unclear.

#### 2.4.4. Analysis Methods

Detailed information on the study sample and therapy characteristics in both the intervention and control groups was systematically obtained and organized. Cochrane’s review data extraction form was utilized to gather trial characteristics. Effect sizes to evaluate the impact of peer support programs in individuals with SMI were determined using Review Manager 5.4.1. software (RevMan). The overall effect size was calculated using the standardized mean difference (SMD), and 95% confidence intervals (CI). A random-effects meta-analysis model considering potential variations in treatment effects across studies and sampling variability was employed for the qualitative analysis.

#### 2.4.5. Tests of Heterogeneity

The limited incorporation of RCTs in our analysis presented difficulties in evaluating publication bias. Typically, researchers utilize methods such as assessing funnel plot asymmetry and performing Egger’s regression test in meta-analyses comprising ten or more studies, as suggested by Dalton in 2016 [35]. However, due to the restricted number of trials (n = 8) included in our study’s quantitative synthesis (meta-analysis), all available techniques for identifying potential publication bias lacked the statistical robustness required for a comprehensive analysis.

## 3. Results

**Figure 1:**
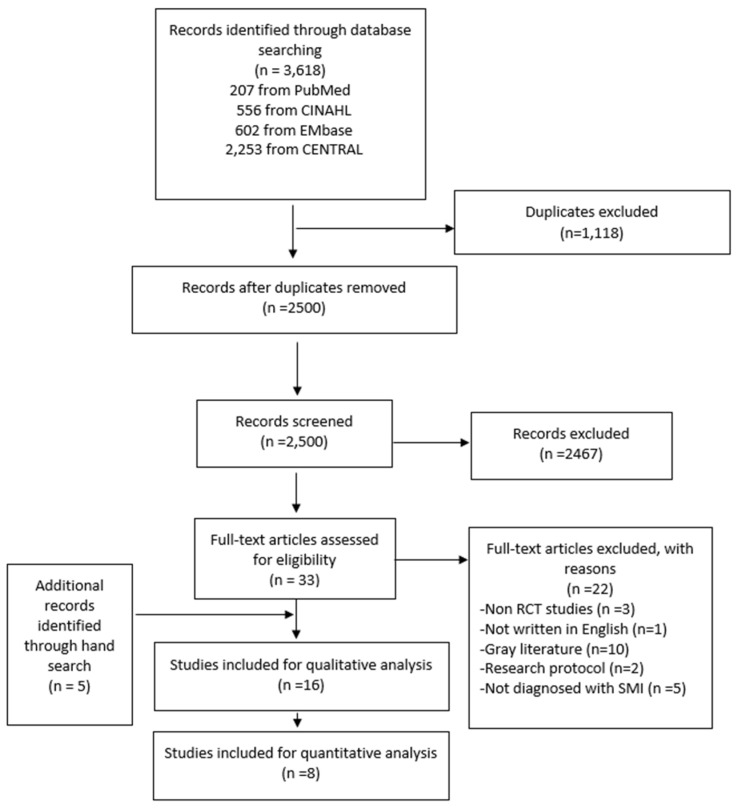
PRISMA flow chart.

The initial search yielded 3618 articles (Figure 1). After eliminating all duplicates, 2500 abstracts and titles in total were reviewed. Among these, 33 RCTs underwent full-text review. In total, 33 full-text RCTs were excluded for various reasons (e.g., not being RCT studies, not being written in English, being gray literature, being research protocols, or not involving individuals diagnosed with SMI). Sixteen RCTs were included for the qualitative analysis, out of which eight met the criteria for meta-analysis and were included in the quantitative analysis.

### 3.1. Characteristics of Included RCTs

Table 1 provides an overview of the key characteristics of the studies included in the review. Sixteen studies [21,23,24,36,37,38,39,40,41,42,43,44,45,46,47,48,49,50,51,52,53], involving 4008 participants in total, met the inclusion criteria. These studies were published over the past 20 years (i.e., from 2003 to 2023) and were conducted across five different countries: the United States (n = 12), the Netherlands (n = 1), the United Kingdom (n = 1), Germany (n = 1), and Australia (n = 1). The sample sizes ranged from 82 to 519 participants. On average, the duration of peer support in the included studies was approximately 8 months (ranging from 8 weeks to 18 months). The mean age of participants ranged from 18 to 75 years, with 45.9% of participants being female. All participants had been diagnosed with SMI, with the most prevalent type being schizophrenia spectrum disorders, across the studies. Nine studies delivered interventions in group mode, while seven delivered interventions one-on-one. Regarding provider supervision, seven trials involved provider supervision during the delivery of peer support, while six trials did not, and three trials did not specify. In total, 14 studies utilized manuals when delivering the program, while 2 studies did not. In all 16 studies, the control group received treatment as usual (TAU).

### 3.2. Study Characteristics of the Measured Outcomes

Table 2 presents detailed outcome measures regarding the effectiveness of peer support programs on individuals with SMI, focusing on major outcomes included in the qualitative synthesis. Two measures of depression were identified: the Center for Epidemiological Studies Depression Scale and Beck Depression Inventory-II. For self-efficacy, the General Self-Efficacy Scale was utilized. Three types of measures of psychiatric symptoms were employed in our review: the Brief Symptom Inventory, the Psychotic Symptom Rating Scale, and the Brief Psychiatric Rating Scale. Measures of relationship were also identified. For empowerment, the Empowerment Scale was used. Lastly, for quality of life, four types of measurements were utilized: the Quality of Life Brief Inventory, the Manchester Short Assessment of Quality of Life, the Quality of Life Scale, and the Quality of Life Instrument Brief Version.

Comparisons of change scores (differences between groups) between those who received treatment and those on the waitlist indicated that the treated group experienced notable enhancements in several aspects, including increased utilization of primary care services, improved quality of the relationship between consumers and physicians, reduced inclination towards seeking emergency or urgent care or avoiding healthcare services altogether, heightened preference for primary care clinics, better detection of chronic health issues, alleviation of pain, and enhanced confidence in self-management of healthcare among consumers.

### 3.3. Risk of Bias

**Figure 2:**
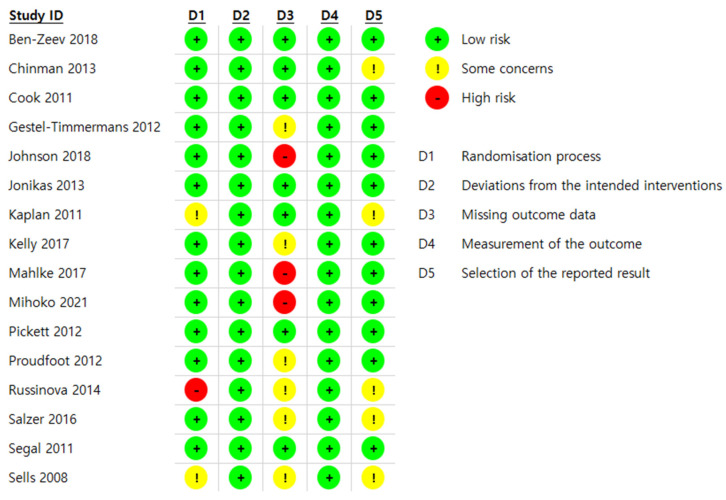
Risk of bias (ROB) [21,23,24,36,37,38,39,40,41,42,43,44,45,46,47,48,49,50,51,52,53].

Of the sixteen RCTs, thirteen studies (81.2%) adequately described their randomiza-tion process. All sixteen RCTs (100%) adequately met the requirements for “deviations from the intended interventions”. Regarding “missing outcome data”, seven RCTs (43.7%) were rated as “low bias”. In the remaining nine RCTs (56%), the description regarding missing outcome data was insufficient, so they were rated as either “high” or “some con-cerns”. In all sixteen (100%), the measurement of outcomes was fully described, so they were rated as “low”. Lastly, regarding “selection of the reported results”, eleven studies (68.7%) were rated as having a “low” risk of bias for the selective reporting assessment (Figure 2).

### 3.4. Effectiveness of Peer Support on Individuals with SMI

**Figure 3:**
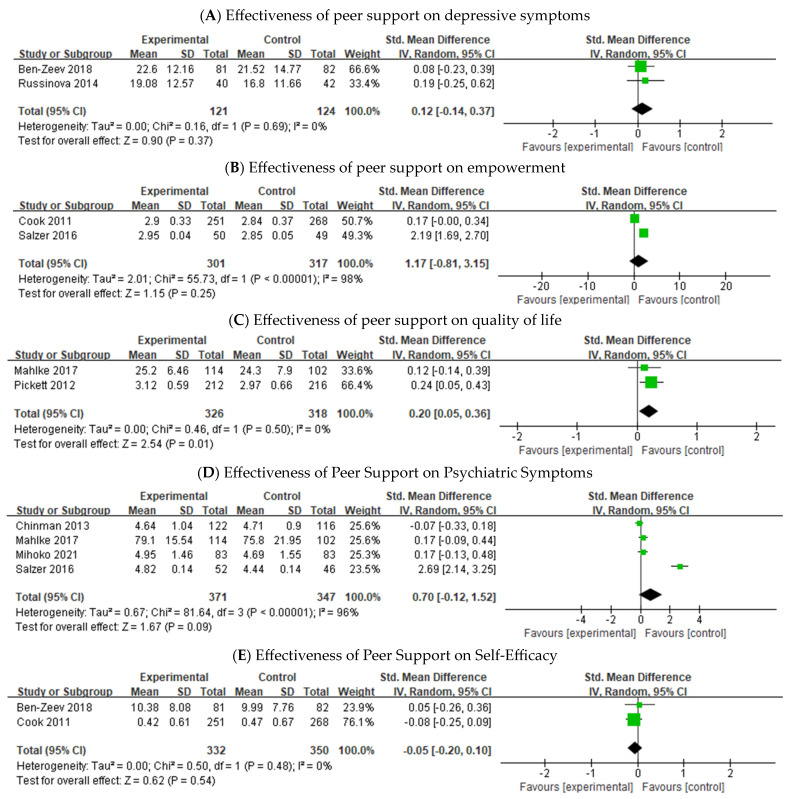
Forest plot of the effectiveness of the peer support program.

Figure 3 displays the forest plots for the effect sizes of peer support programs for individuals with SMI. For depression, two trials were included in the quantitative synthesis, with 245 participants. The pooled SMD of depressive symptoms was d = 0.12; 95% CI, −0.14, 0.37; *p* = 0.37, indicating a small effect size. The Cochran Q test and I^2^ scores for depressive symptoms were Q value = 0.00, *p* = 0.69, I^2^ = 0%, indicating low heterogeneity. The pooled SMD of empowerment was d = 1.17; 95% CI, −0.81, 3.15; *p* = 0.25, indicating a large effect size. The Cochran Q test and I^2^ scores for empowerment (Q value = 2.01, *p* < 0.001, I^2^ = 98%) indicated high heterogeneity. The pooled SMD of quality of life was d = 0.70; 95% CI, −0.12, 1.52; *p* = 0.09, indicating a medium effect size. The Cochran Q test and I^2^ scores for quality of life (Q value = 0.67, *p* < 0.001, I^2^ = 96%) indicated high heterogeneity. The pooled SMD of psychiatric symptoms was d = −0.05; 95% CI, −0.20, 0.10; *p* = 0.54, indicating a small effect size. The Cochran Q test and I^2^ scores for psychiatric symptoms (Q value = 0.00, *p* = 0.48, I^2^ = 0%) indicated low heterogeneity. The pooled SMD of self-efficacy was d = 0.20; 95% CI, −0.05, 0.36; *p* = 0.01, indicating a small effect size. The Cochran Q test and I^2^ scores for self-efficacy (Q value = 0.00, *p* < 0.50, I^2^ = 0%) indicated low heterogeneity.

## 4. Discussion

### 4.1. Findings

Despite the rapid increase in mental illnesses, there remains a shortage of mental health professionals, and societal stigma toward mental health services remains high, impeding continuity and integration of care. For example, in South Korea, despite the existence of policies aimed at deinstitutionalization, the number of psychiatric beds has increased and long-term hospitalization rates remain high [54]. In this context, peer support workers can serve as valuable resources to enhance access to mental health services for individuals with SMI, facilitate connections to community services for those transitioning from hospital to community care, and strengthen continuity of care. Therefore, this study analyzed the effectiveness of peer support programs for individuals with SMI with the goal of examining key strategies that can be applied to peer support program services, including those involved in transitional care programs, in the future.

This review provides an up-to-date summary of research on the effectiveness of peer support programs, a widely utilized mental health care strategy in both inpatient and outpatient settings. We identified 16 RCTs investigating the effectiveness of peer support in individuals with SMI and involving 4008 participants in total from five different countries (the UK, the USA, the Netherlands, Germany, and Australia). The meta-analysis indicated that peer support programs had an overall statistically significant effect in our review, rated as low-risk in quality, with low overall statistical heterogeneity. Most trials, as assessed with a risk of bias evaluation, were rated as having moderate overall quality.

In our meta-analysis, findings from two studies suggested that peer support interventions were statistically significant in improving self-efficacy in individuals with SMI. For example, in Mahlke et al. (2017) [39], the self-efficacy levels in the intervention group who received the peer support program improved by 2.9 points from baseline to the 6-month follow-up, compared with a 1.6-point improvement in the control group. These findings are consistent with a prior meta-analysis [43] examining the effects of self-efficacy in individuals diagnosed with type 2 diabetes (T2D), which found a significant improvement compared with the control group. These results support the idea that self-efficacy can be enhanced through well-designed peer support programs for individuals with SMI. However, more research is warranted to consolidate these results and provide definitive conclusions regarding the effects of peer support programs on self-efficacy in individuals with SMI.

In our review, self-efficacy exhibited a small effect size but demonstrated a significant enhancement in individuals with SMI who participated in the peer support program. This finding aligns with a previous narrative synthesis and meta-analysis [44], which also reported a notable improvement in self-efficacy. Peer-facilitated time-limited group interventions have been shown to yield modest yet significant enhancements in self-efficacy compared with standard treatment protocols. Therefore, it is imperative to develop future peer interventions with a specific focus on enhancing self-efficacy.

In seven (43.7%) out of the 16 RCTs, supervision was included in the peer support program. Having supervision proved effective in terms of program outcomes. Supervision in peer support programs for SMI involves providing oversight, guidance, and support to peer support workers who assist individuals dealing with significant mental health challenges [47]. In future studies, more intervention studies need to be conducted with supervision included rather than solely focusing on peer support alone.

In our review, most study samples included people who had experienced different mental health problems. However, SMI includes various major mental illnesses, each with various signs and symptoms, different effective treatments, and varying levels of effectiveness for peer support [42]. Therefore, future studies need to develop and implement peer support programs tailored to specific mental health problems.

Our review revealed various clinical and psychiatric outcomes. However, none of the studies measured cost-effectiveness. According to previous research [46], peer support programs, either in a group format or one-to-one delivery, are associated with lower overall total healthcare costs during the time period the intervention is being delivered. The cost savings observed were largely due to decreased hospitalization expenses (over 8–12 months in individuals with T2D). Therefore, future studies investigating both one-on-one and group peer support programs for individuals with SMI, especially for long-term benefits, should be conducted.

Our research reveals that, in 11 of the 16 studies, peer support programs were primarily conducted through face-to-face interactions. However, individuals with SMI often lack the motivation to participate in such in-person programs. Moreover, those with SMI frequently lack crucial social support [45]. To make peer support programs more accessible, we suggest exploring alternative methods (e.g., online delivery through standalone applications or web-based platforms) or a combination of both online and face-to-face approaches. Applications can be accessed from anywhere with an internet connection and are particularly beneficial for those in remote areas or with mobility limitations. Online peer support also allows participants to engage at their convenience, accommodating busy schedules or alleviating difficulty attending traditional in-person sessions. Recognizing the changing landscape of support systems, we stress the importance of integrating online and offline methods that reflect current trends. This comprehensive approach fosters community and provides a platform for individuals to connect, build social support networks, and strengthen their social ties.

### 4.2. Limitations

There are several limitations that need to be addressed in this review. First, the pooled analysis included different populations, such as those with schizophrenia, which impacts the generalizability of the findings. When studies aggregate data from diverse populations, the resulting conclusions may not be uniformly applicable to all subgroups within the meta-analysis. Second, out of 16 trials, 12 trials (75%) were conducted in the USA and the remaining ones were carried out in western nations such as the UK, the Netherlands, Germany, and Australia. This geographical distribution might limit the generalizability of the study findings. For example, schizophrenia occurs in approximately 1% of the general population worldwide and is not limited to certain regions. Consequently, the applicability of our results might be restricted to populations sharing similar traits or attributes. Therefore, there is a need for more research studies conducted in various countries to enhance the diversity and generalizability of findings.

Second, 15 trials were conducted in community settings, which might affect the applicability of the findings to other contexts such as inpatient or specialized care settings. Third, due to the restricted number of RCTs available for inclusion, we encountered limitations in our ability to adequately evaluate the presence of publication bias. Standard methods for assessing publication bias, such as examining funnel plot asymmetry and conducting Egger’s regression, are typically recommended for meta-analyses comprising ten or more studies. However, due to the small pool of trials in our analysis, these techniques lacked the necessary statistical power, suggesting potential reporting bias.

Lastly, our search was confined to studies published in English, thus potentially overlooking relevant research conducted in other languages that met the criteria for inclusion in the review. The omission of gray literature may have also heightened the risk of publication bias.

### 4.3. Suggestions

A mixed-methods approach integrating in-depth qualitative research on the experiences between providers and beneficiaries of peer support services with scientific quantitative research could be an effective research strategy. While this study reported only on the impact of peer support services on individuals with SMI, it does not present the effects on the providers, that is, the peer supporters. Research targeting peer supporters has mainly been qualitative in nature. Since peer support services aim to facilitate mutual recovery, there is a need for quantitative studies involving peer supporters to be expanded. Additionally, research should focus on enhancing therapeutic alliances between mental health professionals and peer supporters and collecting evidence to validate the effectiveness of peer support programs.

## 5. Conclusions

In summary, concerning peer support programs for individuals with SMI, we found that they had a statistically significant impact on increasing self-efficacy. Additionally, empowerment showed a large effect size. The overall variability among the trials was low. As our findings suggest, research concerning peer support programs for individuals with SMI is still in its early stages. Our review underscores the need for studies on peer support programs for individuals with SMI, especially those examining self-efficacy outcomes, to encompass a broader geographical scope including more countries and conducted on a larger scale to yield more robust evidence. Additionally, qualitative research studies are needed. Caution is advised when interpreting these findings owing to variations in methodology and the overall quality of the studies. Further clinical trials are required to determine the optimal number of peer support sessions required to effectively support individuals with SMI. At the clinical level, it is advisable to adapt programs to include supervision in the peer support programs for individuals with SMI.

## Figures and Tables

**Table 1:** Descriptive Summary of Included Study Characteristics.

Trials/ Study Design	Country- Recruitment	Diagnosis	Mean Age (Year)	Female (%)	No. of Participants (Intervention/ Control)	Peer Support Intervention				Comparison
						Type	Provider Supervision	Duration/ No. of Sessions	Min/Session	
Sells 2008 [36] RCT	The USA— community	61% SPR 70% Multiple	42	38.7	68/69	One-on-one offline (no manual)	Supervised	12 mos. /N/A	N/A	TAU
Cook 2011 [48] RCT	The USA— community	21% SPR 38% BPD 25% DD	46	65.9	276/279	Group offline (manual)	Unknown	8 wks/8	2.5 h	TAU
Segal 2011 [24] RCT	The USA— community	100% SPR	39	53.9	86/53	One-on-one (manual)	No	8 mos /N/A	N/A	TAU
Kaplan 2011 [42] RCT	The USA— community	100% SPR	47	65.7	200/100	One-on-one online (no manual)	No	12 mos/ N/A	N/A	TAU
Pickett 2012 [49] RCT	The USA— community	39% SPR 40% BPD	43	55.6	212/216	Group (manual)	Unknown	8 wks/ N/A	2.5 h	TAU
Gestel-Timmermans 2012 [50] RCT	The Netherlands— community	33% SPR	44	66.1	168/165	Group offline (manual)	No	12 wks/ weekly	2 h	TAU
Proudfoot 2012 [37] RCT	Australia— community	100% BPD	18–75	69.8	139/134/134	One-on-one online (manual)	No	8 wks/ N/A	N/A	TAU
Chinman 2013 [21] RCT	The USA— community	100% SMI	53	25.5	149/133	One-on-one offline (manual)	Supervised weekly	12 mos/ 8	2.5 h	TAU
Jonikas 2013 [51] RCT	The USA— community	21% SPR 38% BPD 25% DD	older than 18	65.9	276/279	Individual and group offline (manual)	Unknown	2 mos/ weekly	2.5 h	TAU
Russinova 2014 [52] RCT	The USA— community	34% SPR 33% BPD 26% DD	older than 18	68.3	40/42	Group offline (manual)	No	10 wks/ N/A	90 min	TAU
Salzer 2016 [23] RCT	The USA— community	100% SPR	49	46.5	50/49	One-on-one offline (manual)	Supervised	12 mos/ N/A	N/A	TAU
Kelly 2017 [38] RCT	The USA— community	37% SPR 19.2% BPD 39% DD	46	53.6	76/75	One-on-one offline (manual)	Supervised weekly	6 mos/ N/A	N/A	TAU
Mahlke 2017 [39] RCT	Germany— four hospitals	28% SPR 15% BPD 25% DD	42	57.4	114/102	One-on- one offline (manual)	Supervised bi-weekly	12 mos/ 1 meeting per week	1 h	TAU
Ben-Zeev 2018 [53] RCT	The USA— community	49% SPR 28% BPD 23% MDD	49	41.1%	163 (81/82)	One-on-one mobile (manual)	No	3 mos/ weekly	N/A	TAU
Johnson 2018 [40] RCT	The UK— community	14% SPR 12% BPD 23% DD	40	60.1%	441 (220/218)	One-on-one offline (manual)	Supervised bi-weekly	18 mos/ 10	1 h	TAU
Maru 2021 [41] RCT	The USA— community	33% SPR 29% BPD 15% DD	45	51.2%	166 (83/83)	One-on-one and group offline (manual)	Supervised weekly	12 mos/ 23	N/A	TAU

SPR, schizophrenia spectrum disorders; BPD, bipolar Disorder; MDD, major depressive disorder; DD, depressive disorder; TAU, treatment as usual; RCT, randomized controlled trial.

**Table 2:** Study Outcomes.

Trials	Outcomes	Assessments	Measure by Time Point	Result-Key Finding
Sells 2008 [36]	(1) Relationship (2) Addiction severity (3) Quality of life	(1) Barrett-Lennard Relationship Inventory (BLRI) (2) Addiction Severity Index (ASI) (3) Quality of Life Inventory– Brief Version (QOLI-B)	(1) Post-intervention (6 mos) (2) Post-intervention (12 mos)	Participants with peers reported a better therapeutic relationship than the control group at the 6-mo follow-up.
Cook 2011 [48]	(1) Psychiatric symptom (2) Hopefulness (3) Quality of life	(1) Brief Symptom Inventory (BSI) (2) Hope Scale (HP) (3) World Health Organization Quality of Life Brief Instrument (WHOQOL-BREF)	(1) Baseline (2) Post-intervention (8 weeks) (3) Post-intervention (6 mos)	Significantly improved increases in overall clinical symptoms, hopefulness, and quality of life over time compared with the control group
Segal 2011 [24]	(1) Empowerment (2) Self-Efficacy (3) Social integration (4) Psychiatric symptoms (5) Hopefulness	(1) Empowerment scale (2) Self-Efficacy Scale (3) Independent Social Integration Scale (ISIS) (4) Brief Psychiatric Rating Scale (BPRS) (5) Hopelessness Scale	(1) Baseline (2) Post-intervention (8 mos)	Neither psychiatric symptoms nor hopelessness differed by service condition across time.
Kaplan 2011 [42]	(1) Recovery (2) Quality of life (3) Empowerment (4) Medical & social support (5) Psychiatric symptoms	(1) Recovery Assessment Scale (RAS) (2) Quality of Life (3) Empowerment Scale (4) Medical Outcomes Study (MOS) (5) Hopkins Symptoms Checklist (HSCL)	(1) Baseline (2) Post-intervention (4 mos) (3) Post-intervention (12 mos)	No differences between conditions on the main outcomes
Pickett 2012 [49]	(1) Attendance (2) Empowerment (3) Self-efficacy	(1) Attendance Rates (2) 28-item Empowerment Scale (3) Patients Self-advocacy Scale (PSAS)	(1) Baseline (2) Post-intervention (8 wks) (3) Post-intervention (6 mos)	Significant increases in overall empowerment, empowerment-self-esteem, self-advocacy, and assertiveness, with these outcomes improved over time
Gestel-Timmermans 2012 [50]	(1) Hopefulness (2) Quality of life (3) Self-efficacy (4) Empowerment (5) Loneliness	(1) Health Hope Index (HHI) (2) Manchester Short Assessment of Quality of Life (MANSA) (3) Mental Health Confidence Scale (MHCS) (4) Dutch Empowerment Scale (5) Loneliness Scale	(1) Baseline (2) Post-intervention (3 mos) (3) Post-intervention (6 mos)	A significant and positive effect on empowerment, hope, and self-efficacy beliefs but not on quality of life and loneliness; effects of the intervention persisted three months after participants completed the course
Proudfoot 2012 [37]	(1) Perceptions (2) Secondary outcomes: anxiety, depression, work and social adjustment, self-esteem, life satisfaction, health focus of control, stigma	(1) Brief Illness Perception Questionnaire (Brief IPQ) (2) Goldberg Anxiety and Depression Scale (GADS) (3) Work and Social Adjustment Scale (WSAS) (4) Satisfaction with Life Scale (SWLS) (5) Multidimensional Health Locus of Control (MHLC) (6) Mood Monitoring	(1) Baseline (2) Post-intervention (8 weeks) (3) Post-intervention (3 mos) (4) Post-intervention (6 mos)	Increased perceptions of control, decreased perceptions of stigmatization, and improvements in levels of anxiety and depression but no differences between groups on outcomes; adherence to the treatment program was significantly higher than that in the control group
Chinman 2013 [21]	(1) Recovery (2) Quality of life (3) Activation (health self-management efficacy (4) Interpersonal relations (5) Psychiatric symptoms	(1) Recovery Self-Assessment (RSA) (2) Mental Health Recovery Measure (MHRM) (3) Quality of Life Instrument Brief Version (QOLI) (4) Patient Activation Measure (PAM) (5) BASIS-R Scales	(1) Baseline (2) Post-intervention (12 mos)	Improved significantly more than control group, but with no significant differences
Jonikas 2013 [51]	(1) Patient self-advocacy (2) Hopefulness (3) Quality of life (4) Psychiatric symptoms	(1) Patient-Self-Advocacy Scale (PSAS) (2) Hope Scale (HS) (3) Quality of Life Brief Instrument (WHOQOLBREF) (4) Brief Symptom Inventory (BSI)	(1) Baseline (before 6 wks) (2) Post-intervention (6 weeks) (3) Post-intervention (6 mos)	Significantly more engaged in self-advocacy with service providers compared with the control group
Russinova 2014 [52]	(1) Stigma (2) Self-efficacy (3) Recovery (4) Depression	(1) Internalized Stigma of Mental Illness Scale (2) Approaches to Coping With Stigma Scales (3) Personal Growth and Recovery Scale (PGRS) (4) Empowerment Scale (5) Center for Epidemiological Studies Depression Scale (CES-D) (6) Self-Efficacy Scale	(1) Baseline (2) Post-intervention (10 wks) (3) Post-intervention (3 mos)	Significantly reduced self-stigma, greater use of proactive coping with community activism, and perceived recovery and growth
Slazer 2016 [23]	(1) Community Participation (2) Recovery (3) Quality of life (4) Empowerment (5) Working alliance	(1) Temple University Community Participation Measure (2) Recovery Assessment Scale (RAS) (3) Lehman’s Quality of Life (4) Empowerment Scale (5) Working Alliance Measure	(1) Baseline (2) Post-intervention (6 mos) (3) Post-intervention (12 mos)	No differences between groups in outcomes
Kelly 2017 [38]	(1) Health service (2) Satisfaction (3) Self-management confidence (4) Health issues	(1) Health service utilization (2) Satisfaction with primary care provider (3) Self-management attitudes and behaviors (4) Routine health screening (5) Health status (medical diagnosis, pain)	(1) Baseline (2) Post-intervention (6 mos)	Significant improvements in the therapeutic relationship, increased preference for primary care clinics, and improved reductions in pain compared with the control group but increased confidence in consumer self-management of healthcare and decreased preference for emergencies were not significantly higher than the control group
Mahlke 2017 [39]	(1) General self-efficacy scale (GSE) (2) Quality of life (3) Clinician ratings (4) Service use	(1) General Self-Efficacy Scale (GSE) (2) EQ5D (EuroQoL-D5) (3) GAF (Global Assessment Functioning), CGI (Clinical Global Impression) (4) MSLQ-R (Motivated Strategies for Learning Questionnaire)	(1) Baseline (2) Post-intervention (6 mos) (3) Post-intervention (12 mos)	Significantly higher scores of self-efficacy at the 6-month follow-up compared with the control group
Ben-Zeev 2018 [53]	(1) Participant (2) Satisfaction (3) Clinical outcomes	(1) Engagement Rate (2) Satisfaction Rate (3) Symptom Checklist–9 (SCL-9) (4) Beck Depression Inventory-2 (BDI-2) (5) Psychotic Symptom Rating Scales (PSYRATS) (6) Recovery Assessment Scale (RAS) (7) Quality of Life (QoL)	(1) Baseline (2) Post-intervention (3 mos) (3) Post-intervention (6 mos)	Significant improvements in recovery were seen for the control group post-treatment, and significant improvements in recovery and quality of life were seen for the intervention group at 6 mos. Clinical outcomes significantly improved in both groups but did not differ.
Johnson 2018 [40]	Acute care readmission	(1) Client Satisfaction Questionnaire (2) Illness Management and Recovery Scale (3) University of California, Los Angeles (UCLA) Loneliness Scale (4) Lubben Social Network Scale	(1) Baseline (2) Post-intervention (4 mos) (3) Post-intervention (18 mos)	Readmission to acute care within 1 year was significantly lower in the intervention group than in the control group.
Maru 2021 [41]	(1) Vocational and prevocational activity (2) Quality of life (3) Work hope (4) Work readiness (5) Working alliance (6) Participant rate	(1) Vocational and Prevocational Activity (2) Quality of Life (3) Work Hope Scale (4) Work Readiness Scale (5) Working Alliance Inventory-Short Form (6) Participant data of session	(1) Baseline (2) Post-intervention (6 mos) (3) Post-intervention (12 mos)	There were some differences in vocational preparation areas and vocational activities between the intervention and control groups. Some aspects of quality of life and career aspirations improved in the intervention group compared with the control group.

## Data Availability

The data presented in this study are available upon request from the corresponding author. The data are not publicly available due to respondents’ privacy.

## Supplementary Materials

The following supporting information can be downloaded at https://www.mdpi.com/article/10.3390/healthcare12121179/s1, Table S1: Example of full search strategy used in the literature search.
