# Supplementary material for: Effectiveness of Peer Support Programs for Severe Mental Illness: A Systematic Review and Meta-Analysis
Source: Healthcare (Basel). 2024 Jun 11;12(12):1179. doi: 10.3390/healthcare12121179 (PMC11203176; doi:10.3390/healthcare12121179)
Supplement: Supplementary file 1 [file healthcare-12-01179-s001.zip › healthcare-2982528-supplementary.pdf]

**Supplement 1. Example of full search strategy used in the literature search**

MEDLINE via PubMED

| Searches | Results |
|---|---|
| (((((((((((((((((((((((((mental disorder[MeSH Terms]) OR (psychiatric disorder[Title/Abstract])) OR (mentally ill persons[MeSH Terms])) OR (mentally disabled[Title/Abstract])) OR (severe mental illness[Title/Abstract])) ) AND (peer counselor[Title/Abstract])) OR (peer provider[Title/Abstract])) OR (peer educator[Title/Abstract])) OR (peer specialist[Title/Abstract]))) OR (consumer advocator[Title/Abstract])) OR (peer support programs[Title/Abstract])) OR (Psychosocial Interventions[MeSH Terms])) OR (peer-delivered intervention[Title/Abstract])) OR (mutual support[Title/Abstract])) OR (self-help groups[Title/Abstract])) OR (organization[Title/Abstract] AND administration[Title/Abstract]) | 207 |

CINAHL

| Searches | Results |
|---|---|
| SU mental disorders OR TI psychiatric disorder OR AB psychiatric disorder OR TI mentally ill persons OR AB mentally ill persons OR TI mentally disabled AB mentally disabled OR TI severe mental illness OR AB severe mental illness AND TI peer educator OR AB peer educator OR TI peer specialist OR AB peer specialist OR TI consumer advocator OR AB consumer advocator OR TI peer support programs OR AB peer support programs OR SU Psychosocial Interventions OR TI peer-delivered interventions OR AB peer-delivered intervention OR TI self-help groups OR AB self-help groups AND TI organization and administration OR AB organization and administration | 556 |

EMBASE

| Searches | Results |
|---|---|
| 'mentally ill persons':ab,ti OR 'mental disease'/exp OR 'mental disease' OR 'mentally disabled person'/exp OR 'mentally disabled | 602 |

| | |
|---|---|
| person' OR 'severe mental illness'/exp OR 'severe mental illness' OR 'mental patient'/exp OR 'mental patient' AND 'peer counselor':ab,ti OR peer provider':ab,ti OR peer educator' OR peer specialist':ab,ti OR consumer advocator':ab,ti OR peer support programs':ab,ti OR psychosocial interventions':ab,ti OR peer-delivered intervention':ab,ti OR mutual support':ab,ti OR self-help groups':ab,ti OR 'organization'/exp AND 'administration'/exp | |

COCHRANE (CENTRAL)

| Searches | Results |
|---|---|
| ((((((((((((((((((((((((mental disorder[MeSH Terms]) OR (psychiatric disorder[Title/Abstract])) OR (mentally ill persons[MeSH Terms])) OR (mentally disabled[Title/Abstract])) OR (severe mental illness[Title/Abstract])) ) AND (peer counselor[Title/Abstract])) OR (peer provider[Title/Abstract])) OR (peer educator[Title/Abstract])) OR (peer specialist[Title/Abstract]))) OR (consumer advocator[Title/Abstract])) OR (peer support programs[Title/Abstract])) OR (Psychosocial Interventions[MeSH Terms])) OR (peer-delivered intervention[Title/Abstract])) OR (mutual support[Title/Abstract])) OR (self-help groups[Title/Abstract])) OR (organization[Title/Abstract] AND administration[Title/Abstract]) | 2253 |
